# The Nature of 3, 4-Methylenedioxymethamphetamine (MDMA)-Induced Serotonergic Dysfunction: Evidence for and Against the Neurodegeneration Hypothesis

**DOI:** 10.2174/157015911795017146

**Published:** 2011-03

**Authors:** Dominik K Biezonski, Jerrold S Meyer

**Affiliations:** Neuroscience and Behavior Program, University of Massachusetts, Amherst MA 01003, USA

**Keywords:** MDMA, serotonin, neurodegeneration, neurotoxicity, serotonin transporter, vesicular monoamine transporter 2, gene expression, biochemical downregulation.

## Abstract

High doses of the recreational drug 3,4-methylenedioxymethamphetamine (MDMA, “Ecstasy”) have been well-documented to reduce the expression of serotonergic markers in several forebrain regions of rats and nonhuman primates. Neuroimaging studies further suggest that at least one of these markers, the plasma membrane serotonin transporter (SERT), may also be reduced in heavy Ecstasy users. Such effects, particularly when observed in experimental animal models, have generally been interpreted as reflecting a loss of serotonergic fibers and terminals following MDMA exposure. This view has been challenged, however, based on the finding that MDMA usually does not elicit glial cell reactions known to occur in response to central nervous system (CNS) damage. The aim of this review is to address both sides of the MDMA-neurotoxicity controversy, including recent findings from our laboratory regarding the potential of MDMA to induce serotonergic damage in a rat binge model. Our data add to the growing literature implicating neuroregulatory mechanisms underlying MDMA-induced serotonergic dysfunction and questioning the need to invoke a degenerative response to explain such dysfunction.

## INTRODUCTION

The recreational drug 3,4-methylenedioxymethamphetamine (MDMA; “Ecstasy”) is a ring-substituted amphetamine in the phenylisopropylamine family of substances [1]. MDMA is categorized as an “entactogen” primarily due to its empathogenic effects in human users, which accounts for its early clinical use from the late 1960’s through the late 1970’s as an adjunct to psychotherapy [2]. The use of this substance soon spread to the general population, and despite its reported utility in the therapeutic setting, MDMA and the related compound 3,4-methylenedioxyamphetamine (MDA) were given a Schedule 1 designation due to their reputedly high abuse potential, lack of assessed safety for use under medical supervision, and emerging evidence suggesting “neurotoxic” effects of these compounds in animals, particularly on the serotonergic neuromodulatory system in the brain [3, 4]. However, the exact nature of these effects has proven difficult to determine, ineluctably leading to considerable debate on this issue within the drug abuse research community. The most critical areas of disagreement have involved the definition of what constitutes “neurotoxicity”, determination of the mechanism(s) responsible for the observed neurochemical changes following MDMA exposure, and the appropriateness of using certain biochemical andhistological techniques to assess the capacity of this compound to cause neurodegeneration. More than 20 years later, these issues remain unresolved, indicating the need for new approaches to enhance our understanding of the adverse effects of this compound on the serotonergic system.

## EVIDENCE SUPPORTING THE NEURODEGENERATION HYPOTHESIS

The neurodegeneration hypothesis of MDMA (and MDA) neurotoxicity first appeared in the published literature in the mid-to-late 1980s based on a number of studies in rats [5-10] and a few studies in nonhuman primates [3, 11, 12] (note: we are excluding mouse studies from this review because mice exhibit a strong dopamine (DA) neurotoxicity in response to MDMA that is largely absent in rats and monkeys). In most of the rat studies, MDMA was administered either in a single dose ranging from 10 to 40 mg/kg or in multiple doses of 10 or 20 mg/kg MDMA over several consecutive days. “Neurotoxicity” was assessed at 1-2 weeks after the final drug treatment by measuring changes in forebrain levels of serotonin (5-hydroxytryptamine, 5-HT), DA, norepinephrine (NE), and their respective major metabolites or, in some cases, changes in synaptosomal monoamine uptake or radiolabeled transporter binding. These studies consistently revealed major decrements in 5-HT and 5-hydoxyindoleacetic acid (5-HIAA) levels following MDMA exposure, with very modest, if any, effects on levels of the other monoamine neurotransmitters. Similarly, 5-HT uptake and 5-HT transporter (SERT) binding were significantly reduced in response to high-dose MDMA treatment regimens. The extent and persistence of these reductions were subsequently shown to depend on a variety of factors, including dosing regimen, route of administration, which enantiomer of MDMA is administered, and inter-species differences in ADME (absorption, distribution, metabolism, and excretion) profiles [4]. The effect of ambient temperature is also of particular importance, in that exposure to the drug at higher temperatures (>21-23°C) usually causes hyperthermia and an exacerbation of neurotoxic effects, whereas exposure at lower temperatures (<19-21°C) tends to yield a hypothermic response and an attenuation of these effects [13]. At around 21°C, previous findings from our laboratory indicate that the dysregulatory effects of MDMA on body temperature cause some animals to become hyperthermic while others exhibit a hypothermic response [14].

The ability of a high-dose MDMA treatment regimen to produce long-lasting reductions not only in tissue 5-HT and 5-HIAA content but also in 5-HT reuptake and SERT binding is significant because the SERT protein is selectively expressed by serotonergic neurons and is found in the plasma membrane of serotonergic axons and terminals. Consequently, decreases in the amount of SERT (measured either by radioligand binding or using an uptake assay) after MDMA exposure could be indicative of compromised axonal and nerve terminal integrity. In fact, many studies have now demonstrated dramatic decreases in SERT binding following various MDMA dosing regimens and post-administration periods of analysis [4]. Importantly, these analyses have also demonstrated the effect of MDMA on 5-HT and SERT depletion to be region specific. For example, areas such as the striatum, hippocampus, and cortex seem to be affected more strongly than some other areas such as the hypothalamus or certain thalamic nuclei [15].

Some of the most powerful evidence offered in support of the neurodegeneration hypothesis comes from studies involving immunohistochemical (IHC) visualization of serotonergic fibers. Most IHC studies have used antibodies against 5-HT itself, though a few have stained for SERT or tryptophan hydroxylase (TPH), the rate-limiting enzyme in the biosynthesis of 5-HT [16-20]. Early on, researchers reported evidence for transient MDMA-induced swelling and fragmentation of 5-HT-immunoreactive fibers in the rat forebrain followed shortly by a disappearance of many of these fibers [7, 21]. Fine-caliber axons believed to originate from the dorsal raphe nuclei (DRN) seemed to be particularly vulnerable to these effects, in contrast to the larger varicose axons believed to originate from the median raphe nuclei (MRN) that were mainly spared from the drug-induced insult. Not surprisingly, these findings were interpreted as showing that high doses of MDMA rapidly damage ascending serotonergic projections (manifested by the swollen appearance of the fibers at early time points), which is followed by a degeneration of the damaged axons and terminals (manifested by their disappearance at later time points). Importantly, MDMA was not found to decrease the number of 5-HT-immunoreactive cells within the raphe nuclei, suggesting that this compound causes a degeneration of forebrain serotonergic fibers (i.e., distal axotomy) without inducing cellular death.

Long-term studies using either IHC or SERT autoradiography have demonstrated gradual post-MDMA recovery of serotonergic fiber density (based on a reappearance of stained fibers or an increase in SERT binding back to normal levels). Depending on the MDMA dosing regimen, brain area, and species studied, significant (even full) recovery may occur anywhere from 8 weeks to a full year (e.g., see [15, 22]). Nevertheless, studies from Ricaurte’s group have shown incomplete recovery in certain brain areas and/or in particular animals as far out as 12-18 months post-treatment [23, 24]. Another important finding from these time course studies is the late appearance of a serotonergic hyperinnervation of certain subcortical brain areas, a phenomenon thought to reflect axonal/terminal sprouting following synaptic loss [18, 23, 25].

Finally, several other kinds of experimental findings have been offered in support of the neurodegeneration hypothesis. First, there are reports of MDMA-induced damage to axons, terminals, and cell bodies in a few forebrain areas based on well-established markers of neurodegeneration such as silver impregnation and Fluoro-Jade B staining [6, 26, 27, 28]. Second, Callahan and coworkers [29] found a reduction in [^3^H] proline anterograde transport from the raphe nuclei to forebrain structures following MDMA treatment, which could reflect a loss of rostrally projecting serotonergic fibers. Finally, there is substantial evidence that MDMA can provoke significant increases in oxidative stress in the brain, which is one of the proposed mechanisms by which this compound could produce neurodegenerative effects [30, 31]. Nevertheless, caveats can be raised regarding each of these findings. With respect to the above mentioned markers of neurodegeneration, these markers are non-specific and in fact, serotonergic axons are not very sensitive to silver impregnation methods [7]. In addition, the relatively limited distribution of silver staining compared to the widespread loss of 5-HT immunoreactivity throughout much of the rostral forebrain (i.e., cortex, hippocampus, and striatum) raises serious doubts about whether the degenerating axons seen in MDMA-treated animals are actually serotonergic (note also that due to the absence of serotonergic cell bodies in the forebrain, degenerating cells in this region are also presumably non-serotonergic). A similar issue of non-specificity applies to the drug-induced reduction in anterograde transport, since the cell populations within the rostral raphe are known to be highly heterogeneous [32]. Thus, indices of neurodegeneration or of axonal integrity (i.e., anterograde or retrograde transport) need to be bolstered with appropriate immunohistochemical markers of serotonergic neurons to confirm the identity of the degenerating fibers or cells. Indeed, a recent study by Kovács *et al.* [17] found MDMA-induced decreases in serotonergic fiber density assessed by means of either TPH or SERT immunostaining, yet there was no evidence for a blockage of fast axonal transport in the remaining visible TPH-immunoreactive fibers. As a final point, there seems to be little doubt that high doses of MDMA cause increased oxidative stress as well as other consequences that are likely to produce significant cellular dysfunction. What remains unclear, however, is whether these effects occur selectively in serotonergic neurons and whether this ultimately leads to the distal axotomy proposed by the neurodegeneration hypothesis.

## EVIDENCE AGAINST THE NEURODEGENERATION HYPOTHESIS

Although the neurodegeneration hypothesis has dominated the MDMA neurotoxicity field for over two decades, findings have appeared in the literature that are inconsistent with this view. One problematic area concerns whether or not MDMA causes glial responses considered to be characteristic of CNS injury. O’Callaghan and Miller were the first researchers to measure glial responses to MDMA insult. They examined the dose-dependent effects of MDMA in rats on regional levels of glial fibrillary acidic protein (GFAP), an astroglial structural protein previously shown to be elevated as a consequence of CNS damage-induced astrocyte hypertrophy. At doses up to 30 mg/kg given twice daily, there were no increases in GFAP in the cortex, hippocampus, or striatum measured 2 days after the last drug dose, despite massive 5-HT depletions in the same brain areas [33]. Increased GFAP levels were finally observed in the cortex and striatum when the animals were administered 75-150 mg/kg twice daily MDMA for 2 days; however, even at those extreme doses there was no change in GFAP in the hippocampus. A positive control experiment with the established serotonergic neurotoxin 5,7-dihydroxytryptamine (5,7-DHT) demonstrated significant elevations in GFAP levels in all brain areas studied, which coincided with the 5-HT-depleting effects of the treatment. Several later studies by other investigators found a similar lack of effect of “neurotoxic” MDMA doses on measures of either astroglial (GFAP expression or cleaved-tau immunoreactivity) or microglial ([^3^H] PK-11195 binding, OX-6 immunoreactivity, or HSP32 expression) reactivity [34-37], although contrary findings have been reported in several instances [16, 38, 39]. Overall, despite the presence of some inconsistencies in the literature, there is substantial evidence that MDMA doses that produce substantial, long-lasting reductions in 5-HT and other serotonergic markers do not reliably provoke astroglial or microglial responses, thus questioning whether such reductions truly reflect structural damage to the serotonergic system.

Two other recent studies provide evidence that is inconsistent with a loss of serotonergic fibers and terminals following MDMA. Wang *et al.* [40] showed that the apparent loss of forebrain serotonergic projections (determined by immunoautoradiography) following MDMA treatment in rats could be restored when the animals were given the 5-HT precursor 5-hydroxytryptophan (5-HTP) prior to tissue analysis. This finding suggests that serotonergic fibers may remain intact following MDMA exposure, but that MDMA-induced reduction of 5-HT pools within these axons and terminals preclude their detection by immunological staining methods. However, it should be noted that because 5-HTP can be converted to 5-HT in all monoaminergic neurons by the enzyme aromatic L-amino acid decarboxylase, this “restoration” could be due at least partially to staining of 5-HT-containing dopaminergic and/or noradrenergic fibers that were not visualized prior to the 5-HTP treatment. A different approach was taken by Callaghan and coworkers [41], who measured the effects of MDMA on 5-HT clearance *in vivo* in the hippocampal CA3 region using chronoamperometry. Despite significant drug-induced decreases in hippocampal 5-HT content, *in vitro* 5-HT uptake measured in synaptosomal preparations, and SERT binding in the CA3 area, there was no change in 5-HT clearance *in vivo*. Interestingly, p-methoxyamphetamine, another neurotoxic amphetamine, did cause a reduction in *in vivo* 5-HT clearance measured 2 weeks after dosing. Although there are several possible interpretations of the MDMA results, such results again suggest that SERT-expressing axons and terminals in the hippocampus may remain intact following MDMA, although for reasons as yet unknown, expression and functioning of the transporter appear compromised when assessed by standard *in vitro* binding and uptake assays.

## TOWARDS RECONCILING THE NEUROTOXICITY CONTROVERSY

At least some of the debate regarding the nature of MDMA-induced serotonergic dysfunction is due to a lack of consensus regarding the definition of drug-neurotoxicity, specifically a failure to distinguish between substances that merely deplete marker proteins within intact neurons and those that cause measurable neurodegeneration. This is an important distinction to consider since drug-induced neurodegeneration is more likely to cause irreparable consequences for neurobehavioral functioning. Given the plethora of evidence showing the 5-HT- and SERT-depleting effects of MDMA, this substance can certainly be considered “neurotoxic” in terms of causing serotonergic dysfunction. The question at hand, however, is whether serotonergic marker depletion by MDMA is reflective of neurodegeneration or rather is an effect of biochemical downregulation in the absence of tissue damage.

While most investigators in this field equate MDMA-induced reductions in serotonergic markers with a neurodegenerative process, this interpretation can be challenged. For example, although post-MDMA decreases in SERT binding as well as SERT-, TPH-, and 5-HT-immunoreactive fiber density may indeed result from loss of serotonergic fibers, it should be underscored that such analyses depend on the binding of radioligands and antibodies, respectively, to proteins that may be liable to regulation by MDMA (of course, 5-HT is also included here, since its synthesis depends on the presence of active TPH enzyme). For example, as discussed, MDMA causes a depletion of both TPH and 5-HT, perhaps reducing levels of these antigens in IHC studies to below thresholds for detection, thus only giving the appearance of missing fibers. In the long-term, some evidence suggests that SERT gene expression may be negatively regulated by MDMA exposure [42], which could lead to reductions in SERT binding and immunoreactive fiber density in the absence of physical damage. Additionally, since binding assays typically make use of plasma membrane preparations, it is possible that MDMA-induced enhancement of SERT trafficking by endocytosis [43] could lead to decreases in plasmalemmal SERT binding irrespective of altered terminal integrity (although this possibility remains speculative at the present time). As such, it is important to appreciate that MDMA-induced serotonergic deficits can be explained by factors not necessarily dependent on axonal damage.

Recent studies in our laboratory have been aimed at providing new information bearing on the issue of MDMA neurotoxicity. Our first objective was to address a controversy in the literature concerning whether or not “neurotoxic” doses of MDMA lead to reductions in SERT expression by immunoblotting. This controversy arose when two studies by Wang *et al.* [36, 37] found no changes in SERT protein expression in MDMA-treated animals when expression was measured by this method. This surprising result occurred despite substantial drug-induced reductions in 5-HT levels and SERT binding, the typical method for assessing MDMA-related reductions in SERT expression. Subsequently, Xie and colleagues [20] reported decreased SERT protein by immunoblotting following high-dose MDMA treatment, as well as a reduction in the density of SERT-immunoreactive fibers using two different antibodies (one of which was the same as that used for the immunoblotting procedure). In an attempt to help resolve this discrepancy, we conducted our own immunoblotting study of SERT using an approach similar to that of Xie *et al.* [20] in that we performed several positive control procedures to validate our SERT immunoblotting methods prior to assessing the effects of MDMA on expression of this protein. Specifically, by using tissue sources known to contain different amounts of the protein (i.e., parietal cortex and hippocampus from wild-type mice versus SERT-knockout mice; hippocampus from saline- versus 5,7-DHT-treated rats; and various brain regions with differential expression of SERT protein), we screened several different commercially-available anti-SERT antibodies for their ability to yield authentic SERT protein bands as distinguished by the expected changes in band density relative to other, presumably non-specific, bands. Although several of the antibodies tested failed our screening methods (see [44] for details), we did identify one antibody that generated a valid band in the range of ~76 kDa [44], which is consistent with the predicted molecular weight of glycosylated plasmalemmal SERT protein [45]. When we subsequently used this validated procedure to investigate SERT protein expression in whole-tissue lysate preparations 2 weeks following an MDMA binge regimen (4 x 10 mg/kg), expression was profoundly reduced in the striatum, hippocampus, and cortex [44]. These findings are consistent with the extensive radioligand binding literature discussed earlier showing that high doses of MDMA lead to large decreases in SERT in a number of different forebrain areas. 

We next asked whether or not MDMA similarly affected the expression of two other proteins present in serotonergic nerve terminals, namely TPH and the vesicular monoamine transporter 2 (VMAT-2). For this experiment, we chose to study synaptosomal preparations instead of whole tissue lysates, and TPH and VMAT-2 immunoblotting methods were again validated by appropriate control procedures. The use of VMAT-2 as a marker for potential MDMA-induced terminal loss was particularly advantageous for two reasons. First, as a vesicle-specific protein, VMAT-2 is highly concentrated in monoamine nerve terminals. Second and more importantly, studies on the dopaminergic system have shown a loss of VMAT-2 in terminal fields (i.e., striatum) both following administration of a DA neurotoxin such as methamphetamine (METH) [46] and in Parkinson’s disease where the DA nerve terminals have undergone massive degeneration [47]. Thus, the additional quantification of VMAT-2 allowed us to determine whether MDMA-induced changes in SERT levels are accompanied by changes in an established marker of terminal integrity [48]. A similar approach was used by Guilarte and coworkers [49] to distinguish between axotomy versus neuronal plasticity in different brain regions following METH treatment. Of course, a significant limitation in using VMAT-2 as an index of serotonergic terminal density is the additional presence of this protein in noradrenergic as well as dopaminergic synaptic vesicles. To minimize this problem, we adopted the combined approach of (1) focusing on the hippocampus since this structure has a relatively sparse dopaminergic innervation (and therefore only a minor DA contribution to VMAT-2) [50], and (2) lesioning the ascending noradrenergic projections from the locus coeruleus to the forebrain (including the hippocampus) by pretreating some of the animals with N-(2-chloroethyl)-N-ethyl-2-bromobenzylamine (DSP-4), a known NE neurotoxin [51]. Rats were pretreated with DSP-4 or saline and then given our standard MDMA binge treatment regimen or saline 1 week later. Two weeks following the MDMA binge, all animals in the MDMA-treated groups showed substantial reductions in SERT immunoreactivity in striatal, hippocampal, and cortical synaptosomes compared to saline-treated controls. These results corroborated our previous findings of MDMA-induced SERT reductions in whole-tissue lysates. Interestingly, however, we failed to find a significant effect of MDMA on synaptosomal VMAT-2 expression in any brain area of any treatment group, including the hippocampus of DSP-4 pretreated animals (the condition that offered the greatest sensitivity to detecting a loss of serotonergic terminals using the present experimental approach) [44]. Additionally, we were surprised to find that synaptosomal TPH protein levels were completely unaltered in the hippocampus of MDMA-treated compared to control animals (Biezonski and Meyer, manuscript in preparation). These findings strongly suggest that decreases in SERT protein expression following high-dose MDMA exposure may occur in the absence of major terminal loss, presumably as a consequence of regulatory changes induced by the drug.

To investigate a possible mechanism by which MDMA might reduce SERT levels in the absence of axonal damage, we subsequently used quantitative RT-PCR to determine the effects of this compound on SERT (as well as VMAT-2 and TPH-2) gene expression in pooled dorsal and median raphe tissue punches. Importantly, both nuclei project serotonergic fibers to the hippocampus [32], and both contain very few catecholaminergic cell bodies that would be expected to contribute to VMAT-2 gene expression in the tissue samples [32, 52]. Two weeks following the same MDMA binge regimen used in the immunoblotting studies, we found a striking, 50-fold reduction in SERT gene expression within the dorsal/median raphe, with smaller albeit significant (10-15-fold) reductions in expression of both the VMAT-2 and TPH-2 genes [44]. Although not conclusive, these data nonetheless strongly suggest that MDMA-induced downregulation of SERT gene expression may underlie the reductions in SERT protein (measured either by immunoblotting or radioligand binding) consistently seen following treatment with this compound. Furthermore, the reduction in VMAT-2 and TPH-2 gene expression argue against the possibility that compensatory upregulation of gene expression (as a consequence of partial fiber loss) may have normalized protein expression of these markers in our immunoblotting analyses, again supporting the notion that SERT protein depletion resulting from MDMA exposure may indeed occur independent of axotomy.

Finally, in order to more directly determine whether MDMA exposure causes 5-HT synaptic loss, we investigated whether an MDMA binge regimen alters the quantity of serotonergic nerve terminals in the hippocampus 2 weeks following drug exposure. This was accomplished by preparing synaptosomal fractions and then using flow cytometry to measure the number of particles that were double-labeled for synaptosomal-associated protein of 25 kDa (SNAP-25, a general presynaptic terminal marker) and TPH in MDMA- and saline-treated animals. Although flow cytometry is primarily used to characterize and quantify cells according to their size, granularity, and expression of fluorescently-labeled antigens, several groups have shown that the same approach can be used to characterize and quantify synaptosomes obtained from rodent or human brain samples (see, for example, [53-56]). We were surprised to find that the quantity of serotonergic synaptosomes defined as mentioned above not only was not decreased in the MDMA group, but actually showed a significant increase relative to controls (Biezonski, Lu, and Meyer, manuscript in preparation). These results suggest that MDMA may cause an acute reactive synaptogenesis which, in turn, may help account for the long-term hyperinnervation of certain brain regions previously observed in some MDMA-treated animals [18, 23, 25]. Nevertheless, given the novelty of our findings and the current absence of corroborative studies, it is important to caution that our data should at most be considered preliminary evidence for an acute effect of MDMA on serotonergic synaptogenesis. Other studies are needed to investigate this issue using other experimental approaches, including quantification of additional relevant markers such as synaptophysin [57], growth-associated protein of 43 kDa (GAP-43, [58]), or brain-derived neurotrophic factor [59]. Taken together, based on the discrepancy between the present results and previously published findings involving immunostaining for 5-HT, SERT, or TPH, we question the use of standard immunohistochemical techniques to address the neurodegenerative potential of MDMA, unless investigators can stain for a serotonergic neuron-specific antigen whose expression is shown to be unaffected by high-dose MDMA treatment regimens.

The major implication of our findings is that MDMA-induced 5-HT marker depletion may not necessarily result from a degenerative response. Nevertheless, given that all of our methods for detecting MDMA neurotoxicity in the present experiments were indirect (i.e., relied on quantifying protein/gene expression of markers liable to regulation) and measured at only one time point, our findings do not prove unequivocally that the effects of MDMA on serotonergic nerve terminals occur in the absence of neurodegeneration. They do, however, exemplify the dramatic effects this compound can have on the regulation of several serotonergic markers, thus questioning the need to invoke distal axotomy as the only explanation for MDMA-induced serotonergic dysfunction. As such, future studies should aim to confirm our findings by the use of more direct measures not dependent on marker staining. Such experiments should investigate ways to directly highlight and subsequently measure changes in serotonergic fiber density in response to MDMA, such as through the use of anterograde or retrograde tracers, or by using reporter genes (e.g., green fluorescent protein) driven by promoters active only in 5-HT neurons.
